# *Caenorhabditis elegans* SET1/COMPASS Maintains Germline Identity by Preventing Transcriptional Deregulation Across Generations

**DOI:** 10.3389/fcell.2020.561791

**Published:** 2020-09-22

**Authors:** Valérie J. Robert, Andrew K. Knutson, Andreas Rechtsteiner, Steven Garvis, Gaël Yvert, Susan Strome, Francesca Palladino

**Affiliations:** ^1^Laboratory of Biology and Modeling of the Cell, Ecole Normale Supérieure de Lyon, CNRS, Université Claude Bernard de Lyon, Université de Lyon, Lyon, France; ^2^Department of Molecular, Cell and Developmental Biology, University of California, Santa Cruz, Santa Cruz, CA, United States

**Keywords:** transgenerational, *elegans*, SET1, transcriptomics, cell identity, germline

## Abstract

Chromatin regulators contribute to the maintenance of the germline transcriptional program. In the absence of SET-2, the *Caenorhabditis elegans* homolog of the SET1/COMPASS H3 Lys4 (H3K4) methyltransferase, animals show transgenerational loss of germline identity, leading to sterility. To identify transcriptional signatures associated with progressive loss of fertility, we performed expression profiling of *set-2* mutant germlines across generations. We identify a subset of genes whose misexpression is first observed in early generations, a step we refer to as priming; their misexpression then further progresses in late generations, as animals reach sterility. Analysis of misregulated genes shows that down-regulation of germline genes, expression of somatic transcriptional programs, and desilencing of the X-chromosome are concurrent events leading to loss of germline identity in both early and late generations. Upregulation of transcription factor LIN-15B, the C/EBP homolog CEBP-1, and TGF-β pathway components strongly contribute to loss of fertility, and RNAi inactivation of *cebp-1* and TGF-β/Smad signaling delays the onset of sterility, showing they individually contribute to maintenance of germ cell identity. Our approach therefore identifies genes and pathways whose misexpression actively contributes to the loss of germ cell fate. More generally, our data shows how loss of a chromatin regulator in one generation leads to transcriptional changes that are amplified over subsequent generations, ultimately leading to loss of appropriate cell fate.

## Background

Preserving germ cell identity is essential for fertility and the passage of genetic information from one generation to the next. In *Caenorhabditis elegans*, loss of germ cell identity has been observed in multiple experimental contexts. Differentiated somatic cells were initially observed in the gonads of animals lacking conserved translational regulators MEX-3 and GLD-1 (Ciosk et al., 2006). Subsequently, forced expression of a master regulatory protein was shown to convert germ cells into somatic cells following RNAi knock-down of genes encoding histone chaperones (*lin-53* or *hmg-3*), Polycomb Repressive Complex 2 (PRC2) subunits, or overexpressing GLP-1/Notch (Tursun et al., 2011; Patel et al., 2012; Seelk et al., 2016; Kolundzic et al., 2018). In addition, altering H3K4 methylation levels in the germline (Katz et al., 2009; Käser-Pébernard et al., 2014; Robert et al., 2014), mutations in nuclear RNAi pathways (Weiser et al., 2017; Rogers and Phillips, 2020), or depletion of germ granules (called P granules in *C. elegans*) (Updike et al., 2014; Knutson et al., 2017) were shown to induce spontaneous loss of germline identity. The observation that multiple factors and pathways protect germline identity underlines the complexity of regulatory networks involved in this process. How these interact with each other remains to be established.

Expression profiling experiments have shown that P-granule components, PRC2, the H3K4 histone methyltransferase SET-2, and GLP-1/Notch signaling regulate germline transcriptional programs, and their deregulation is associated with decreased expression of germline genes and derepression of somatic genes (Gaydos et al., 2012; Robert et al., 2014; Seelk et al., 2016; Knutson et al., 2017). Deregulation of germline-specific transcriptional programs therefore plays an essential role in loss of germline identity. These experiments also revealed that multiple regulatory mechanisms converge on common sets of genes, as shown for the antagonistic roles played by PRC2/MES-4 and GLP-1/Notch signaling in regulating gene expression from the X and maintaining germ cell identity (Seelk et al., 2016).

Although the above studies have been useful in describing the transcriptional profiles appropriate for maintenance of a functional germline, they could not distinguish between genes whose misregulation directly contributes to loss of germline identity from those whose misregulation is a secondary consequence of this loss. In this study, the progressive loss of germline identity in animals lacking the SET1/COMPASS homolog SET-2 allowed us to compare transcriptional profiles from early generation mutant germlines, in which fertility is minimally compromised, to late-generation fertile and sterile animals (Li and Kelly, 2011; Xiao et al., 2011). Based on this analysis, we describe a transcriptional signature of the process leading to loss of germ cell fate and sterility.

Our data shows that progressive deregulation of the same transcriptional networks, both at early generations when loss of germline identity is primed, and at later generations nearing sterility, contributes to loss of germ cell fate. We identify downregulation of germline genes, derepression of somatic programs and desilencing of the X-chromosome as direct contributors to loss of germline identity. At the level of individual genes, ectopic expression of the LIN-15B transcription factor, the CCAAT/enhancer-binding proteins (C/EBP) homolog CEBP-1, and components of the TGF-β signaling pathway all show progressive deregulation as animals near sterility (Ceol et al., 2006; Yan et al., 2009; Savage-Dunn and Padgett, 2017; Gumienny and Savage-Dunn, 2018). RNAi inactivation of genes whose expression increases over generations, including *cebp-1* and TGF-β/Smad signaling components, was able to delay the onset of sterility, showing they individually contribute to maintenance of germ cell identity. Altogether, these results identify downstream effectors of SET-2/COMPASS that play an active role in preserving a functional germline, and provide novel insight on how heritable changes in gene expression can lead to loss of cell identity.

## Results

### Loss of Germline Identity in *set-2* Mutants Correlates With Progressive and Widespread Transcriptional Deregulation

**FIGURE 1:**
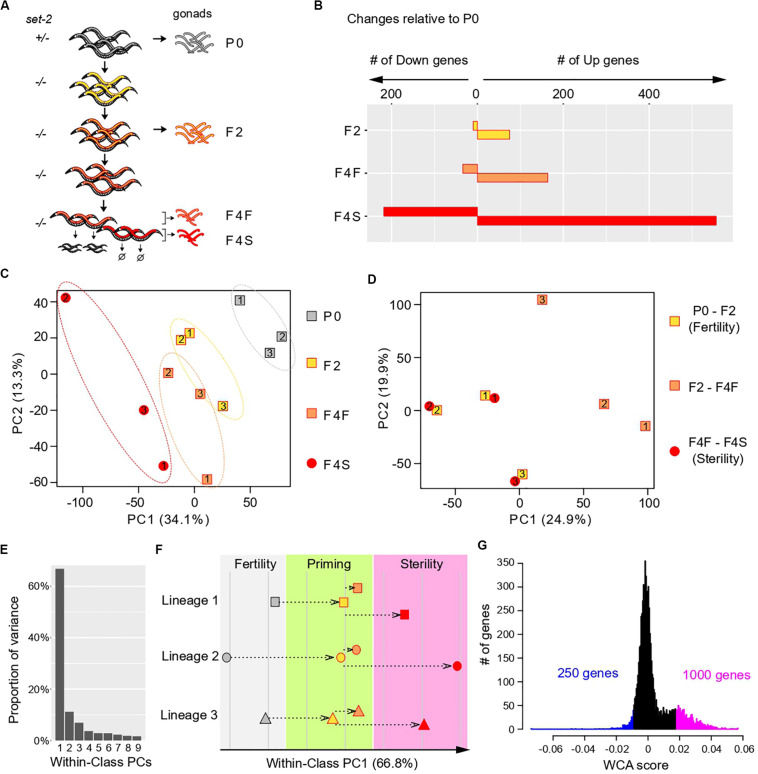
Multivariate analysis of transgenerational transcriptional changes in *set-2* mutant germlines as they progress to sterility. **(A)** Experimental design of the strategy used to determine transcriptional changes that take place in the gonads of *set-2* mutant animals as they progressively lose their germline identity. Gonads were dissected from P0 *set-2(*+)/*set-2(–)* hermaphrodites, from *set-2(–)*/*set-2(–)* hermaphrodites, and from fertile and sterile F4 *set-2(–)*/*set-2(–)* hermaphrodites for RNA-seq analysis. **(B)** Number of genes showing differential expression as compared to P0 at a conservative statistical threshold (adjusted *p* < 10e-6). **(C)** Principal Component Analysis (PCA) on expression levels. Circles and squares correspond to samples and are numbered according to their lineage. **(D)** PCA performed on expression differences between samples of the same lineage. Circles and squares correspond to the indicated pairwise differences, numbers indicate lineages. **(E–G)** Results of Within-Class Analysis (WCA) obtained by considering each lineage as a class. **(E)** Proportion of within-class variance explained by each component. **(F)** Coordinates of samples on the first component (one symbol per lineage, same color scheme as in **C**). **(G)** Histogram showing the distribution of gene coefficients on the first components. Genes having extreme coefficients (colored in blue and magenta) are the major contributors to the orientation of the first component. These coefficients are referred to as “WCA scores” in the main text.

We raised *set-2/*+ heterozygous animals at 25°C (P0 generation) to obtain *set-2* fertile F1 homozygotes that we then grew for one additional generation (F2). F2 animals were allowed to self-fertilize and produce progeny for two additional generations, and F4 progeny were scored and separated in two distinct sets: F4 fertile and F4 sterile. Note that although within each generation the fertility of individual animals is highly variable (Supplementary Figure S1A), by the F6-F8 generation all animals become sterile. We carried out germline dissections followed by RNA sequencing (RNA-seq) on three separate lineages started from *set-2*/+ single mothers (Figure 1A).

We first used the RNA-seq data sets to identify changes in gene expression across generations, considering lineages as independent replicates and comparing F2, F4 fertile, and F4 sterile animals to the parental samples (P0). This confirmed that loss of germline identity in *set-2* mutants is associated with transcriptional deregulation (Robert et al., 2014), and showed that the number of misregulated genes progressively increased between P0, F2, F4 fertile, and F4 sterile animals (Figure 1B). In all generations, we observed significantly more upregulated than downregulated genes, as previously reported for healthy germlines from *set-2* animals raised at 20°C (Robert et al., 2014). Furthermore, the total number of misregulated genes was approximately three times higher in sterile than in fertile F4 animals, suggesting that sterility is associated with an increase in transcriptional deregulation over time.

### Transcriptomic Deregulation Occurs in Two Distinct Steps

To visualize how changes in gene expression progress over subsequent generations in *set-2* mutant germlines, we applied Principal Component Analysis (PCA) on the expression matrix of all genes (Figure 1C). As expected from our initial analysis, the distance on the plot relative to the parental samples increased over generations and was highest for samples from F4 sterile animals. In addition, we observed a significant dispersion of the 3 lineages: inter-lineage distance was more pronounced between F4 fertile samples than between P0 or F2 samples, and was highest for F4 sterile samples (Figure 1C, dotted ellipses).

The divergence between samples indicates that for each lineage, the set of misregulated genes is largely distinct, and prompted us to look for a transcriptional signature common to all lineages across generations. For all genes within each lineage we computed the difference in expression (log ratios) between P0 and F2 samples (early changes), F2 and F4 fertile samples (later changes that partially compromise fertility), and F4 fertile and sterile samples (changes associated with fully penetrant sterility). Strikingly, for each of the three lineages PCA on the resulting matrix revealed clustering between P0 and F2 samples and F4 fertile-F4 sterile samples (Figure 1D). By contrast, F2-F4 fertile sets mapped separately in the PCA plot. This provided a key insight: for each of the 3 lineages, a set of genes is misregulated in early generation (P0 to F2), through a process we define and refer to hereafter as “priming,” and further misregulated in the same direction (up or down) in sterile F4 animals.

### Deregulation of a Specific Set of Genes Defines a Path to Sterility

We next investigated whether a common set of genes undergoes the 2-step deregulation process described above in all 3 lineages: misregulation in P0-F2, and further misregulation in F4 fertile-F4 sterile, in the same direction. We first discarded genes that were poorly expressed in all samples, since their variation may simply be due to background signal, and focused our analysis on highly expressed genes (see “Materials and Methods”). This left a reduced set of 7,238 genes (Supplementary Figure S1C and Supplementary Table S1). We then considered each lineage as an independent class and performed Within-Class Analysis (WCA), a derivative of PCA that takes into account variation due to a given factor (lineage in this case) (Baty et al., 2006). The first component of WCA captured 67% of the Within-Class variation (Figure 1E). Plotting samples along within-class PC1 revealed that transcriptional deregulation progressed in a continuum across generations in all 3 lineages, first during the priming step (P0-F2), and then toward sterility (from F2 to F4 sterile) (Figure 1F). Based on these results, we conclude that genes that contribute to PC1 define a path to sterility. To identify genes that vary along this path, we plotted the distribution of the coefficients that define PC1, which we called “WCA scores” (Figure 1G). In this analysis, negative WCA scores are attributed to genes globally downregulated, and positive scores to genes globally upregulated across generations. Consistent with a bias toward upregulation, WCA scores were asymmetrically distributed, with significantly more genes having a positive score. The most extreme WCA scores (positive or negative) identify genes that contribute the most to PC1, and hence to sterility.

We next examined how transcription deregulation progressed in each lineage: six of the nine genes with the highest negative WCA score were downregulated between P0 and F2 animals, and eight were more strongly downregulated between F2 and sterile F4 than between F2 and fertile F4 (Supplementary Figure S2). Similarly, eight of the nine genes with the most positive WCA score were upregulated between P0 and F2, and all were more strongly upregulated between F2 and sterile F4 than between F2 and fertile F4 (Supplementary Figure S3). Therefore, our WCA analysis identified genes with similar transcriptional trajectories in all three lineages. We arbitrarily set negative and positive thresholds, and hereafter refer to the genes with the largest negative and positive scores as “downregulated contributors” (250 genes) and “upregulated contributors” (1000 genes), respectively (Figure 1G and Supplementary Table S1). These genes make the largest contributions to within-class PC1, and their deregulation is tightly associated with the loss of germline function in all 3 lineages. We collectively refer to these genes as “contributors.” Note that by definition all “contributors” first become deregulated in the priming event. Interestingly, we found a significant overlap between our list of contributors and genes differentially expressed in *set-2* mutant germlines at 20°C (Supplementary Figure S4 and Supplementary Table S2)^1^. Because at 20°C transgenerational loss of fertility is only observed after 15–20 generations (Xiao et al., 2011; Herbette et al., 2017), these results suggest that the misregulation of these same genes may also contribute to sterility at 20°C, and heat-stress accelerates this process.

### Repression of Germline Genes and Expression of Somatic Programs Contribute to Loss of Fertility in *set-2* Mutant Germlines

**FIGURE 2:**
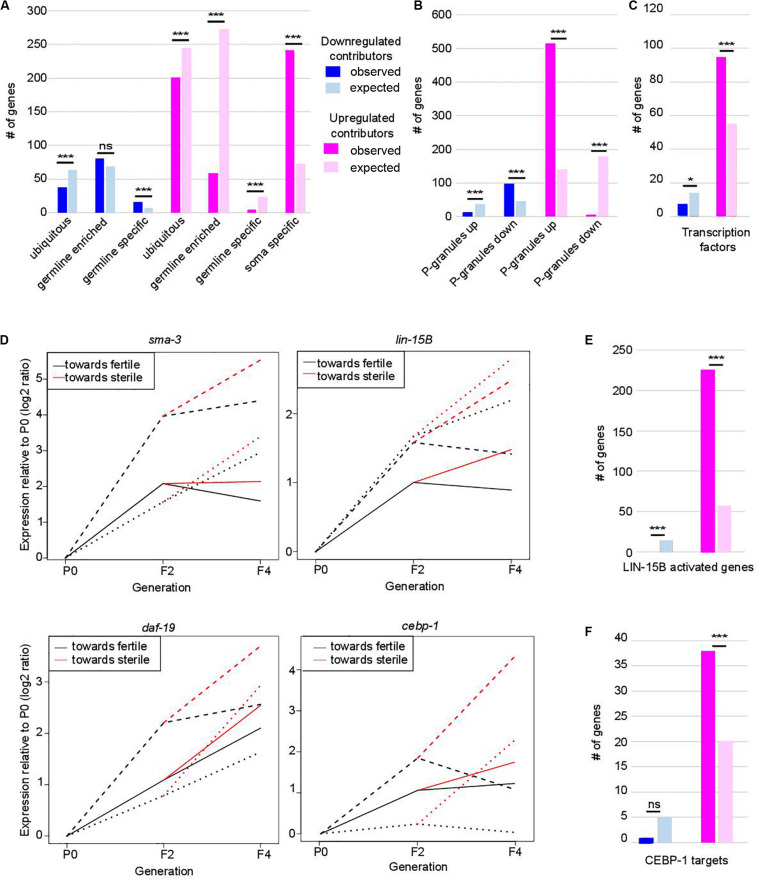
Functional classification of contributors to *set-*2 dependent loss of germline identity. In **(A–C,E,F)** *p-value < 0.05 and ***p-value < 0.001 calculated by hypergeometric test. **(A)** Classification of contributors according to their expression profiles in wild-type animals. Lists of down- and upregulated contributors were compared to lists of ubiquitous, germline-enriched, germline-specific, and soma-specific genes defined in (8). Downregulated contributors are depleted for ubiquitous genes (*p* = 2.3e-4) and enriched for germline-specific genes (*p* = 2e-4); upregulated contributors are depleted for ubiquitous genes (*p* = 1.3e-4), germline-enriched genes (*p* = 2.41e-119), and germline-specific genes (*p* = 2.41e-119), and enriched for soma-specific genes (*p* = 2.96e-78). **(B)** Comparison of lists of contributors with genes up- or downregulated in the gonads of adult worms in which P granules had been depleted by RNAi for 2 generations [PG(–); 8]. Color code for down- and upregulated contributors is the same as in **(A)**. Downregulated contributors are depleted for genes upregulated in PG(–) (*p* = 5.5e-06), and enriched in genes downregulated in PG(–) (*p* = 1.1e-15); upregulated contributors are depleted for genes downregulated in PG(–) (*p* = 1.4e-90), and enriched in genes upregulated in PG(–) (*p* = 1.4e-218). **(C)** Comparison of contributors with transcription factors (list wTF3.0). Color code for down- and upregulated contributors is the same as in **(A)**. *p* = 0.03 for under-enrichment of downregulated contributors in transcription factors and *p* = 1.2e-8 for over-enrichment of upregulated contributors in transcription factors. **(D)** Relative expression to P0 of *sma-3*, *lin-15B*, *daf-19*, and *cebp-1* transcripts in F2, fertile F4 and sterile F4. The three independent lineages analyzed are represented by distinctive line formats. For each gene, expression relative to P0 increases more when F4 animals became sterile than when they remain fertile. **(E)** Comparison of contributors with LIN-15B-activated genes. Color code for contributors is the same as in **(A)**. *p* = 3.4e-7 for under-enrichment of downregulated contributors in LIN-15B-activated genes and *p* = 1.7e-93 for over-enrichment of upregulated contributors in LIN-15B-activated genes. **(F)** Comparison of contributors with CEBP-1 targets. Color code for contributors is the same as in **(A)**. *p* = 0.2 for under-enrichment of downregulated contributors in CEBP-1 targets and *p* = 4.8e-5 for over-enrichment of upregulated contributors in CEBP-1 targets.

Loss of germ-cell identity in *C. elegans* germlines depleted of PRC2 or P-granule components is associated with decreased expression of germline genes and increased expression of somatic genes (Gaydos et al., 2012; Knutson et al., 2017). Comparison of our lists of contributors with published lists of gene expression profiles in wild-type animals revealed a similar trend in *set-2* mutants, suggesting that this transcriptional reprogramming is an early priming event (Figure 2A and Supplementary Table S1; Knutson et al., 2017). Downregulated germline-specific contributors include genes required for meiosis (*apc-10*, *him-5*), the *wago-4* argonaute-encoding gene, and genes that encode P-granule components (*cey-2*, *cey-3*, and*pgl-3*).

Progressive loss of fertility in *set-2* mutants is associated with loss of P-granules (Robert et al., 2014). We found that *set-2* inactivation and loss of P-granule components share similar transcriptional signatures (Figure 2B), with a significant overlap for both down- and up-regulated contributors (Supplementary Table S2; *p*-value = 1.11e-15 and 1.18e-219, respectively). Commonly downregulated genes are enriched in both ubiquitous and germline genes (Supplementary Table S2; *p*-value = 2.4e-4 and 5.4e-6), while commonly upregulated genes show no particular bias (Supplementary Table S2). Notably, of the 240 soma-specific genes that are upregulated contributors in *set-2* mutant germlines, only 133 are also upregulated in P-granule-depleted germlines (Supplementary Table S2; *p*-value = 0.07). Therefore, SET-2 and P-granules appear to have both common and unique functions in the repression of somatic gene expression in the germline.

Strikingly, we found that 389 of the 941 transcription factors found in *C. elegans* [wTF3.0; (Fuxman Bass et al., 2016)] were present in the list of 7,238 genes analyzed by WCA, and of these 101 were identified as contributors (1.5-fold enrichment; *p* = 6e-06) (Figure 2C and Supplementary Table S3). These include the R-SMAD SMA-3, the THAP transcription factor LIN-15B, the RXF family transcription factor DAF-19, 7 CEH homeodomain transcription factors, 22 Nuclear Hormone Receptors (NHRs), and (CEBP-1/C/EBP) (Savage et al., 1996; Swoboda et al., 2000; Antebi, 2006; Ceol et al., 2006; Yan et al., 2009). These genes consistently displayed increased expression between P0 and F2 (in the priming step) and a further increase between F2 and sterile F4 (Figure 2D). Significantly, downstream targets of LIN-15B and CEBP-1 were also identified as upregulated contributors, including 226 of the 452 genes activated in PGCs following ectopic expression of LIN-15B (Figure 2E, *p*-value = 1.71e-93; Supplementary Table S3; Lee C. S. et al., 2017), and 38 of 112 CEBP-1 target genes (Kim et al., 2016; Figure 2F, *p*-value = 4.8e-05; Supplementary Table S3). Targets of transcription factors *somi-1* and R06C1.6 were also significantly enriched in the list of upregulated contributors [*p*-value = 0.006 and 0.0002, respectively (Fuxman Bass et al., 2016)], consistent with deregulation of a broad class of transcriptional networks driving loss of germline identity.

### Progressive Upregulation of X-Linked Genes Is Tightly Linked to Onset of Sterility and Loss of Germline Identity in *set-2* Mutant Germlines

**FIGURE 3:**
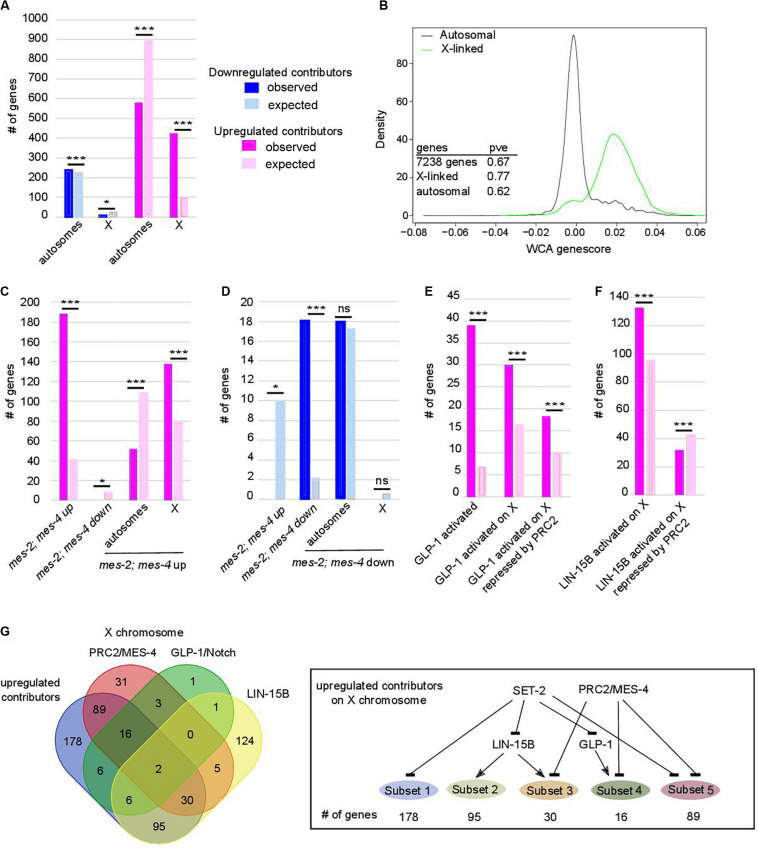
Chromosome X desilencing is a priming event for loss of germline identity. In **(A,C–F)** *p-value < 0.05 and ***p-value < 0.001 calculated by hypergeometric test. Color code in bar graphs is the same throughout. **(A)** Chromosome distribution of contributors. *p* = 4.4e-5 and 6.1e-193 for distribution of down- and upregulated contributors, respectively. **(B)** Distribution of WCA score according to location of the genes on autosomes or the X chromosome (pve = percent of variance explained by PC1 when within-class PCA is run on the indicated set of genes only). **(C)** Comparison of upregulated contributors with *mes-2*; *mes-4* up- and downregulated genes and chromosome distribution of upregulated class. *p* = 2.34e-97 for over-enrichment in *mes-2*; *mes-4* upregulated genes, *p* = 3.6e-4 for under-enrichment in *mes-2*; *mes-4* downregulated genes, *p* = 3.2e-21 for chromosome distribution. **(D)** Comparison of downregulated contributors with *mes-2*; *mes-4* up- and down-regulated genes and chromosome distribution of downregulated class. *p* = 3.9e-5 for under-enrichment in *mes-2*; *mes-4* upregulated genes, *p* = 5.84e-14 for over-enrichment in *mes-2*; *mes-4* downregulated genes, *p* = 0.5 for chromosome distribution. **(E)** Comparison of upregulated contributors with genes activated by GLP-1. *p* = 1.25e-24 for over-enrichment in GLP-1 activated genes, *p* = 7e-6 for over-enrichment in X-linked GLP-1 activated genes, *p* = 1e-2 for over-enrichment in X-linked GLP-1 activated genes repressed by PRC2/MES-4. **(F)** Comparison of upregulated contributors with genes activated by LIN-15B. Color code is the same as in **(A)**. *p* = 7.7e-9 for over-enrichment in X-linked LIN-15B-activated genes, *p* = 8e-3 for over-enrichment in X-linked LIN-15B-activated genes repressed by PRC2/MES-4. **(G)** Comparison of X-linked upregulated contributors with genes repressed by PRC2/MES-4 and genes activated by GLP-1/NOTCH or LIN-15B. Summary of connections between the regulatory networks that regulate various classes of X-linked genes in *set-2* mutant germlines.

Further analysis revealed that upregulated contributors are highly enriched on the X chromosome (Figure 3A, *p*-value = 6e-193). These X-linked genes have a significantly higher WCA score than autosomal genes (Figure 3B), showing that they strongly contribute to sterility and loss of germline identity. PRC2 components MES-2 and MES-6 cooperate with MES-4 to repress the X chromosome and maintain germline identity (Xu et al., 2001; Bender et al., 2004, 2006; Rechtsteiner et al., 2010; Gaydos et al., 2012). Comparison of genes misregulated in the absence of both *mes-2* and *mes-4* (*mes-2;mes-4* double mutants) and our lists identified 188 commonly upregulated genes (Figure 3C; hypergeometric *p*-value = 2.34e-97, Supplementary Table S4), mostly located on the X chromosome (137 genes, hypergeometric *p*-value = 3.2e-21). Conversely, of the 18 commonly downregulated genes, all are found on autosomes (Figure 3D; hypergeometric *p*-value = 5.84e-14). This is unlikely to reflect regulation of *mes-2* or *mes-4* expression levels by SET-2, since neither genes was identified by WCA. Similarly, *set-2* is not a transcriptional target of PRC2 (Gaydos et al., 2012). Rather, our results suggest that PRC2/MES-4 and SET-2 act in parallel pathways to silence the X chromosome and promote proper expression of a subset of autosomal genes.

### Ectopic Expression of GLP-1/Notch Signaling and LIN-15B Contribute to Derepression of X-Linked Genes in *set-2* Mutant Germlines

Polycomb Repressive Complex 2-dependent repression of the X chromosomes in the germline is counteracted by increased GLP-1/Notch signaling: in worms bearing the gain-of-function allele *glp-1(ar202)*, increased signaling induces expression of specific genes normally repressed by PRC2 (Seelk et al., 2016). We found that *glp-1* is over-expressed in all 3 lineages across generations (WCA score = 0.0054), and that GLP-1/Notch targets are upregulated contributors (39 genes, representing a 5.65-fold enrichment; *p*-value = 1.25e-24, Figure 3E). Of these targets, 30 are located on the X chromosome (*p*-value = 7.5e-06, Supplementary Table S1), and 18 of these are upregulated in germlines lacking both MES-2 and MES-4 (*p*-value = 1e-3). For these genes, SET-2 is likely to act redundantly with PRC2/MES-4 through inhibition of GLP-1/Notch (Figure 3G). An additional subset of upregulated contributors on the X chromosome is enriched in targets of the LIN-15B transcription factor (Figure 3F; *p*-value = 7.71e-9), and depleted for PRC2/MES-4 targets (*p*-value = 7e-3). Therefore, SET-2 likely represses their expression through inhibition of LIN-15B and independently of PRC2. Altogether, these data suggest that SET-2 acts upstream of transcription factors and regulatory networks that control X-chromosome repression.

### RNAi-Mediated Inactivation of Upregulated Contributors Delays the Onset of Sterility

So far our analysis identified a set of genes whose deregulation is associated with loss of fertility and germline identity in all 3 lineages analyzed. We reasoned that if increased expression of upregulated contributors is an essential step leading to sterility and loss of germline identity, then decreasing their expression by RNAi over subsequent generations should delay the onset of sterility.

**FIGURE 4:**
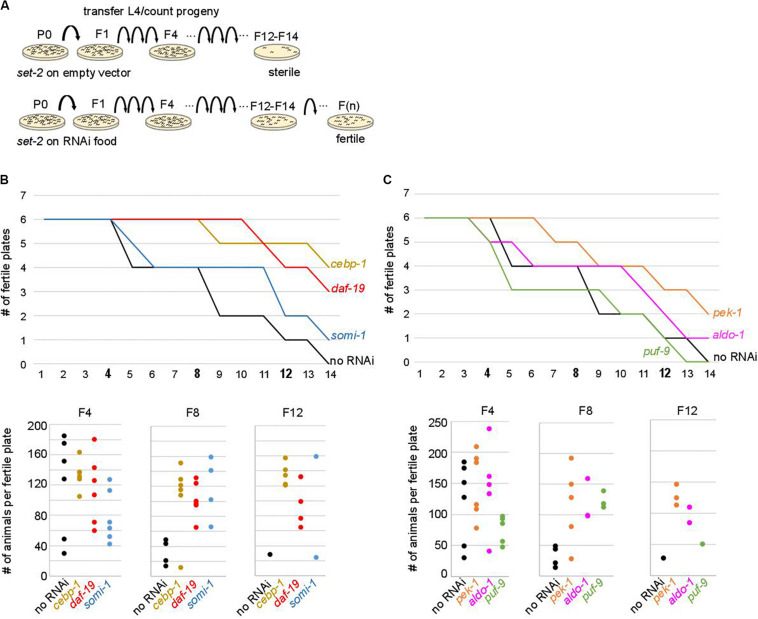
Onset of sterility is delayed by RNAi knock-down of upregulated contributors. **(A)** Experimental design of the RNAi strategy used to test the effect of knock-down of upregulated contributors in *set-2* dependent loss of germline identity. **(B)** *cebp-1, daf-19*, and *somi-1* RNAi knock-down delays the onset of sterility compared to animals fed with an empty RNAi vector (no RNAi). Number of fertile plates (with less than 20 animals) per generation **(upper panel)** and number of animals per fertile plate in F4, F8, and F12 **(lower panel)** are shown. **(C)** *pek-1* and *aldo-1* but not *puf-9* RNAi knock-down result in a delay in the onset of sterility compared to animals fed with an empty RNAi vector (no RNAi).

To test the above hypothesis, *set-2* mutants at the L4 larval stage were transferred to RNAi feeding plates at 25°C, and fertility scored in adults. In control experiments (no RNAi), the total number of progeny dramatically decreased between F4 and F8, and animals became completely sterile at the F14 generation (Figure 4). The delay in the onset of sterility compared to previous experiments (F4-F7) and (Xiao et al., 2011; Robert et al., 2014) is most likely due to the *E. coli* food source (HT115 versus OP50 routinely used for culturing), consistent with bacterial diet influencing fertility (Watson et al., 2015; Heestand et al., 2018). RNAi knock-down of 9 out of 29 upregulated contributors we tested, including the transcription factors *cebp-1*, *daf-19, attf-5*, and *somi-1*, significantly and reproducibly delayed the onset of sterility in *set-2* mutant animals (Figures 4B,C and Supplementary Table S5A). Loss of fertility and brood size were extremely variable, as previously reported for other mutants showing transgenerational loss of fertility (Yanowitz, 2008; Robert et al., 2014). In late generations, delayed onset of sterility was reflected in a larger number of plates with progeny (fertile plates), as well as an increase in the number of animals per fertile plates. For instance, when animals were grown on RNAi plates targeting *cebp-1*, most plates (5/6) contained more than 120 animals at the F12 generation. By contrast, only a single control plate (no RNAi) with less than 30 animals was recovered at the F12 generation (Figure 4B). RNAi knock-down of 11 additional genes (see legends Supplementary Table S5A) resulted in early sterility that prevented further investigation of their role (data not shown). Finally, for the remaining nine genes (*flh-2*, *gfi-3*, *nhr-48*, *jmjd-3.1*, *utx-1*, *kgb-1*, *puf-9*, *miz-1*, and *ncam-1)*, no significant transgenerational effect on fertility was observed after RNAi treatment. For these genes, the absence of an effect may be due to reduced efficacy of the RNAi treatment, or redundancy.

### TGF-β Pathway Components Are Expressed in *set-2* Mutant Germlines, and Contribute to Transgenerational Loss of Fertility

**FIGURE 5:**
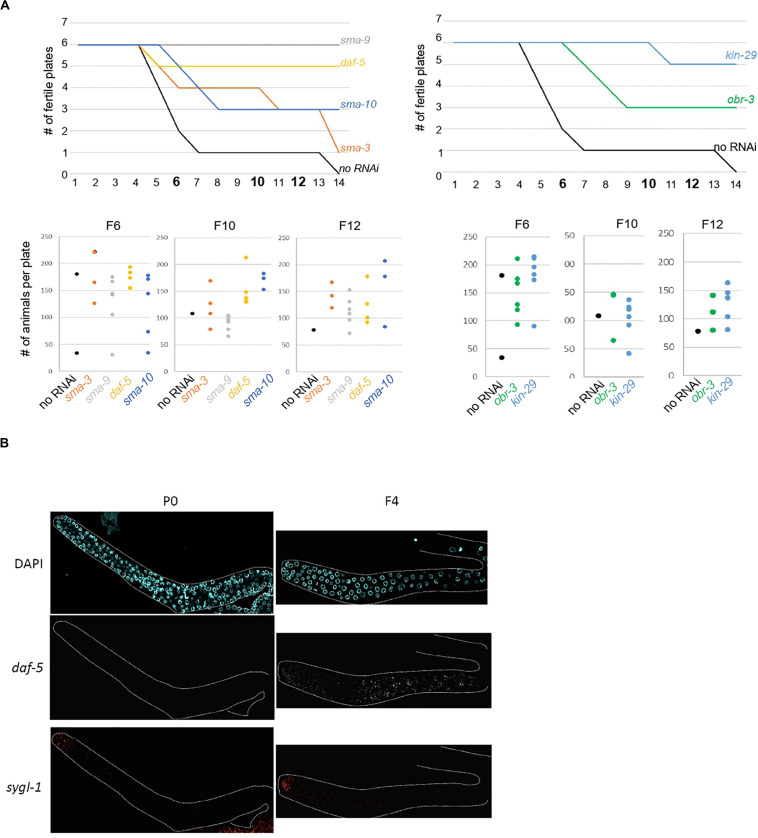
Ectopic TGF-beta signaling contributes to loss of germline identity in *set-2* mutant animals. **(A)** *sma-9*, *daf-5*, *sma-10*, and *sma-3* RNAi knock-down **(left panel)** and *kin-29* and *obr-3* RNAi knock-down **(right panel)** delay the onset of sterility of *set-2* mutant animals compared to animals fed with an empty RNAi vector (no RNAi). Number of fertile plates per generation **(upper panel)** and number of animals per fertile plate in F6, F10, and F12 **(lower panel)** are shown. **(B)** Detection by smFISH of *daf-5* (upregulated contributor) and *sygl-1* (control gene) transcripts in P0 *set-2*/+ and F4 *set-2*/*set-2* germlines. The distal tip of the gonad is on the left.

The above experiments show that our approach successfully identified genes whose increased expression in the germline over generations plays an active role in preserving germline immortality. Because we identified SMA-3 SMAD as an upregulated contributor to loss of germ-cell fate, we asked whether activation of TGF-β signaling in *set-2* mutant germlines actively contributes to loss of germ cell fate. Two canonical TGF-β signaling pathways have been described in *C. elegans*, defined by the DAF-7 and DBL-1 ligands that function through distinct receptors and their pathway-specific SMADs (Supplementary Figure S5A; Savage-Dunn and Padgett, 2017; Gumienny and Savage-Dunn, 2018). Analysis of the WCA list revealed the presence of components of both pathways, including the Type I receptor DAF-1, R-SMADs (SMA-3, DAF-8, and DAF-14), Co-SMADs (SMA-4 and DAF-3), and downstream transcription factors (SMA-9 and DAF-12). By extending the list to include genes with the top 1500 (instead of 1000) WCA scores, we also identified the Type II receptor DAF-4, the extracellular regulator SMA-10, and the transcription factor DAF-5 (Supplementary Table S1). Additional upregulated contributors associated with TGF-β signaling include the KIN-29 serine/threonine kinase (Maduzia et al., 2005) and OBR-3 (Sugawara et al., 2001). Remarkably, for all of these, transcript levels increased in all 3 lineages between P0 and F2 (Figure 2D and Supplementary Figure S5B). Furthermore, for at least 2 of the 3 lineages, expression showed a more significant increase between F2 and F4 sterile than F2 and F4 fertile animals. RNAi knock-down of *sma-3*, *sma-9*, *daf-5*, *sma-10*, *kin-29*, and *obr-3* in *set-2* mutants resulted in a significant and reproducible delay in the onset of sterility (Figure 5A and Supplementary Table S5B), consistent with ectopic activation of TGF-β signaling actively contributing to loss of germ cell fate.

To confirm by an independent assay that components of TGF-β signaling are upregulated in the germline, we performed single molecule RNA-FISH (smFISH) on dissected gonads (Ji and van Oudenaarden, 2012; Lee C. S. et al., 2017). In agreement with our RNA-seq data, *daf-5*, and *sma-3* transcripts were detected in most germ cells of F4, but not P0 animals (Figure 5B and Supplementary Figure S6A).

In wild-type animals, TGF-β signaling regulates multiple pathways, including somatic development, oocytes quality, and germline proliferation [(Ren et al., 1996; Foehr et al., 2006; Luo et al., 2009; Dalfo et al., 2012; Pekar et al., 2017) (Savage-Dunn and Padgett, 2017; Gumienny and Savage-Dunn, 2018)] through downstream target genes. To test whether downstream components of TGF-β signaling are misexpressed in *set-2* mutant germlines, we compared our list to published lists of TGF-β target genes at different developmental stages (Luo et al., 2010). To our surprise, upregulated contributors are enriched in genes upregulated in oocytes from *sma-2* mutant animals, in which the pathway is inactive (Luo et al., 2010), as well as a largely non-overlapping set of genes upregulated in *sma-2* mutant animals at the L4 stage, prior to oocyte development (Supplementary Figures S6B,C). A total of 137 upregulated contributors are genes normally repressed by TGF-β signaling in oocytes of wildtype animals (*p*-value = 5.00e-08), or in L4 (*p*-value = 1.6e-32). This set of upregulated contributors is also enriched in soma-specific genes and LIN-15B activated genes (Supplementary Figure S6D), suggesting that LIN-15B and TGF-β may cooperate at a subset of loci to promote gene expression during loss of germ cell fate. Altogether, these results suggest a complex network whereby ectopic activation of TGF-β pathway components in the germline alters expression of target genes by mechanisms other than canonical signaling.

## Discussion

Protecting the germ cell transcriptional program is essential for germ cell identity and the transmission of genetic information across generations. In this study we used transgenerational expression profiling of dissected germlines to identify genes whose misregulation in the absence of the SET-2/COMPASS H3K4 methyltransferase contributes to transgenerational sterility. We show that altered expression of these contributor genes occurs in a two-step process: an early priming step in which their misregulation has a limited impact on fertility, and a later step in which their expression undergoes further alteration in the same direction (up or down), leading to sterility. smFISH experiments confirmed an increase in transcript levels across most germ cells, consistent with progressive transcriptional changes across the entire population. Our results suggest that the absence of SET-2 results in an increased tendency for somatic genes to be expressed in individual germ cells, and the percentage of germ cells misexpressing these genes increases over generations, eventually compromising fertility. How loss of SET-2 results in the derepression of somatic genes remains unknown. In different organisms including *C. elegans*, loss of COMPASS subunits results in both activation and repression, with no evidence for a direct role in transcription (Lenstra et al., 2011; Clouaire et al., 2012, 2014; Margaritis et al., 2012; Weiner et al., 2012; Beurton et al., 2019).

Our RNA-seq analysis showed important differences in the transcriptomic profiles from different lineages. WCA allowed us to take into account this variability and extract a common transcriptional signature associated with progression to sterility. RNAi inactivation of a total of 9 out of 29 upregulated contributors tested by this approach significantly delayed the onset of sterility in *set-2* mutant animals, as did RNAi inactivation of all of the 6 TGF-β pathway components we tested. Therefore, our approach successfully identified relevant targets. Our findings are consistent with germline mortality being actively regulated by multiple pathways that individually contribute to fertility.

Previous studies focusing on transcriptional deregulation associated with germline loss of identity could not distinguish between genes whose altered expression is a priming event or a consequence of loss of germline identity (Gaydos et al., 2012; Seelk et al., 2016; Knutson et al., 2017). Most notably, loss of germline identity in PRC2/MES-4 mutant backgrounds correlates with derepression of the X chromosome in the germline, but whether this alone is sufficient to cause sterility remains unknown. In the present work we have shown that priming involves concurrent upregulation of somatic genes and downregulation of germline genes, and that upregulated genes are greatly enriched on the X chromosome. We found that affected genes on the X fall into two classes: a large class consisting of genes repressed by SET-2 independently of PRC2, and a smaller class repressed by SET-2 and PRC2/MES-4. The second class supports a common regulatory role for SET-2 and PRC2/MES-4 on the X chromosome and is consistent with studies showing that depletion of SET-2 enhances the sterility of *mes* mutants (Xu et al., 2001). Altogether, our data suggest that derepression of X linked genes may be a primary event driving loss of germ cell identity in different contexts.

Because active chromatin marks including H3K4me3 are mostly absent from the X chromosomes in proliferating and early meiotic germ cells (Kelly et al., 2002; Tabuchi et al., 2018; Han et al., 2019; Kaneshiro et al., 2019), loss of H3K4me3 in *set-2* mutant germlines is unlikely to play a causal role on the X. Rather, loss of H3K4me3 may indirectly affect chromatin structure, and hence transcription on the X. Consistent with such a model, inactivation of SET1/COMPASS subunits results in global changes in repressive H3K9 methylation in both yeast and worms (Robert et al., 2014; Lee et al., 2019; Greenstein et al., 2020) and alters chromatin organization [(Herbette et al., 2017) (see text footnote 1)].

We observed significant overlap between genes misregulated in the absence of P-granule components, and genes that contribute to the onset of sterility in *set-2* mutants. This and the observation that *set-2* mutant germlines nearing sterility lose P-granules (Robert et al., 2014) suggest a role for *set-2* in stabilizing P-granule components. It has been suggested that upregulation of spermatogenesis genes contributes to loss of fertility in P-granule depleted animals (Campbell and Updike, 2015; Knutson et al., 2017). Because their expression is not affected in *set-2* mutants, misregulation of spermatogenesis genes in the absence of P-granules may not be the only contributor to loss of germline identity in these mutants. We note that almost half of upregulated contributors associated with loss of germline identity in *set-2* mutants, including the majority of TGF-β pathway components, are not misregulated in the absence of P granules, further suggesting that the role of SET-2 in maintaining germline identity is not restricted to regulating P granule components.

Transcription factors are over-represented amongst upregulated contributors, consistent with the central role of transcription factor networks in reconfiguring cellular identity (Takahashi and Yamanaka, 2016; Stadhouders et al., 2019). We identified both CEBP-1 and known targets as significant contributors in the loss of cell identity. In *C. elegans* CEBP-1 is required for adult sensory axon regeneration and neuronal stress responses, while mouse C/EBP proteins regulate cell proliferation and differentiation (Nerlov, 2007) and enhance reprogramming of B cells, at least in part by increasing chromatin accessibility to reprogramming factors (Xie et al., 2004; Bussmann et al., 2009; Sadahira et al., 2012; Di Stefano et al., 2014). CEBP-1 expression in the germline could therefore prime cells to respond to additional transcription factors, leading to the neuronal differentiation observed in *set-2* mutant germlines (Robert et al., 2014).

The fact that neither one of the two known TGF-β ligands, DAF-7 and DBL-1, is present in our list of contributors suggests that either *set-2* mutant germline respond to signaling cues from somatic tissues, as reported for mitotic cells in wildtype germlines (Dalfo et al., 2012; Pekar et al., 2017), or that TGF-β signaling is initiated independently of ligand binding in these germlines (Massagué, 2012). An unexpected finding was that expression of TGF-β signaling components in the germline of mutant animals is associated with increased expression of genes normally repressed by the TGF-β pathway in the soma and oocytes of wildtype animals (Luo et al., 2010). Furthermore, TGF-β pathway components were identified as upregulated contributors to sterility, and their down-regulation by RNAi delayed the onset of sterility, indicating that they actively contribute to this process. Several factors may account for this apparent paradox. First, SMAD signal transducers that mediate the downstream transcriptional response to TGF-β signaling have weak affinity for DNA (Massagué, 2012; Hill, 2016). Their transcriptional role therefore depends on robust interaction with other transcription factors and chromatin-associated proteins, resulting in context-dependent cellular responses that may be substantially different between normal, healthy somatic tissues and mutant germlines (Derynck and Zhang, 2003; Massagué et al., 2005; Ikushima and Miyazono, 2010). Second, signaling mechanisms other than the canonical Sma/Mab TGF-β pathway that depends on ligand binding may be activated in the context of *set-2* mutant germlines undergoing loss of germ cell fate, resulting in different outputs (Savage-Dunn and Padgett, 2017; Nickel et al., 2018; Gowripalan et al., 2020). Finally, it is also conceivable that the loss of SET-2 interferes with the repressive function of a SMAD.

More generally, our finding that decreased expression of upregulated contributors to loss of germ cell identity, including *cebp-1* and TGF-β pathway components, is sufficient to delay the onset of sterility, suggests that these genes individually contribute to the process leading to sterility. Therefore, loss of germ cell fate is driven by misexpression of several different genes, whose expression is repressed in wild-type germlines by SET-2.

Our observation that global changes in histone modifications in *set-2* mutant fertile germlines precede the transcriptional changes leading to complete sterility (Robert et al., 2014) is consistent with a role for histone modifications in priming cells for differentiation, and the observation that alterations in genome-wide chromatin organization are generally much more widespread than initial changes in gene expression during the early phases of loss of cell identity (Koche et al., 2011). In the future it will be informative to correlate the transcriptional reprogramming described here to changes in gene expression in individual germ cells.

## Materials and Methods

### Strains and Maintenance

Nematode strain maintenance was described previously (Brenner, 1974). The strain *set-2*(*bn129*) III/*qC1*(*qIs26*) used in this study is described in Herbette et al. (2017). Strain *set-2*(*bn129*)/*qC1*(*qIs26*) was maintained at 20°C and *set-2*(*bn129*)/*qC1*(*qIs26*) animals were switched to 25°C one generation before starting experiments. During the experiments, animals were maintained at 25°C.

### Transcriptomic Analysis

Gonads from *set-2* homozygous worms raised at 25°C? were collected from 3 independent lineages derived from *set-2*(*bn129*)/*qC1*(*qIs26*) [P0 *set-2*(+)/*set-2*(−)] animals by initially picking 25 *set-2*(+)/*set-2*(−) L4 worms and then cloning single worms from subsequent generations to individual plates. Fertility of each worm was assessed before dissection by monitoring egg laying for 24 h after young adult stage. Worms that laid any live progeny were considered as fertile. For the F4 sterile samples, only worms lacking visible embryos in the uterus were collected. For each replicate, seventy five to one-hundred gonad arms were dissected from *set-2*(*bn129*)/*qC1*(*qIs26*) [P0 *set-2*(+)/*set-2*(−)] and *set-2*(*bn129*)/*set-2*(*bn129*) [F2 and F4 *set-2*(−)/*set-2*(−)] animals. Dissected gonads were cut at the gonad bend with 30 1/2-gauge needles in egg buffer (pH 7.3, 27.5 mM HEPES, 130 mM NaCl, 2.2 mM MgCl_2_, 2.2 mM CaCl_2_, and 0.528 mM KCl) containing 0.5% Tween 20 and 1 mM levamisole and collected into Trizol. Total RNA was extracted and ribosomal RNA was depleted using an NEBNext rRNA Depletion Kit (Human/Mouse/Rat) (catalog number E6310). Libraries were constructed using an NEBNext Ultra RNA Library Prep Kit for Illumina sequencing (catalog number E7530) and sequenced at the Vincent J. Coates Genomics Sequencing Laboratory at the University of California, Berkeley, using Illumina HiSeq 2500 and 4000 platforms. For differential expression analysis, raw sequences were first mapped to transcriptome version WS220 using TopHat2 (Kim et al., 2013). Only reads with one unique mapping were allowed, otherwise default arguments were used. Reads mapping to ribosomal RNAs were then removed. HTSeq (Anders et al., 2015) was used to build a count table of expression levels per transcript. DESeq2 (Love et al., 2014) was used to normalize read counts across samples (Supplementary Figure S1B), leading to matrix M that contained the normalized expression levels of 20261 genes. DESeq2 was also used to determine genes for which expression at a downstream generation differed from expression at P0, with no consideration for lineage-dependent relations (Figure 1C). *P*-values were adjusted for multiple testing using the Benjamini–Hochberg method as implemented in DESeq2.

### Multivariate Analysis

We did all further multivariate analysis using R version 3.4.4^2^. To perform PCA (Figure 1C), we first transformed M by applying x = Log_2_(x + 1) to all of its values, obtaining matrix LM, which we transposed and processed with the prcomp() function. To perform PCA on expression differences (Figure 1D), we constructed a 20261 × 9 matrix (DLM), where rows were genes and columns were expression differences of interest (e.g., F2-P0 lineage 1) which we computed by subtracting one column of LM (e.g., F2 lineage 1) from another (e.g., P0 lineage 1). We then transposed DLM and processed it with the prcomp() function. Before applying WCA, we first discarded genes that were poorly expressed in all samples, because their variation may simply be due to varying background signal. To determine a meaningful threshold of minimal expression, we plotted the distribution of all genes according to (i) their maximal expression level in all samples and (ii) their standard deviation of expression across all samples, which could be high due to either meaningless background signal variation or meaningful biological differences between samples. The resulting 2-dimensional density plot revealed 2 subpopulations of genes (Supplementary Figure S1C): a major population that was poorly expressed in all samples, and a secondary population with high expression in at least one sample. For the poorly expressed genes, standard deviation increased monotonously with their maximal expression level, as expected for background variation. A different pattern was observed for highly expressed genes (maximal expression greater than 5). First, their standard deviation of expression was not correlated with maximal expression level. This is expected if change in expression is dependent on SET-2 loss and not on background variation. Second, standard deviation was low for the majority of these genes and high for a small subset. This observation is expected if SET-2 acts only on a subset of genes. We therefore chose an arbitrary cutoff of minimal expression that discarded the first subpopulation (red line in Supplementary Figure S1C), leaving a reduced set of 7,238 genes having at least one value greater than 5 (Supplementary Table S1). We then processed the resulting matrix with the dudi.pca() function of the ade4 package [63] (version 1.7-6)^3^ using parameters scan = FALSE, scale = FALSE, and nf = 4. We then processed the resulting object with the ade4:wca() function using lineages as classes and parameters scan = FALSE, nf = 2. The first column of attribute c1 of the resulting object corresponded to the WCA scores reported in text and figures.

### RNAi Screen

Bacterial clones containing RNAi feeding vectors were collected in the *C. elegans* RNAi collection (made by J. Ahringer, Source Bioscience). The molecular sequence of insert present in each RNAi clones was checked by sequencing (primer ggtcgacggtatcgataagc) after PCR amplification (single primer in duplicated T7 promoter taatacgactcactataggg) performed directly on colonies. For feeding, bacterial clones were amplified 18 h at 37°C in LB complemented with Ampicilline (50 μg/ml). Transcription (from duplicated T7 promoters) was induced by adding IPTG (1 mM final) and growing liquid cultures for 2 additional hours at 37°C. 200 μl of induced cultures were plated on NGM plates complemented with IPTG (1 mM). At each generation, 6 L4 animals were transferred on RNAi plates and grown at 25°C for 3–4 days. Progeny was briefly counted every two generations to evaluate animas fertility. Animals were considered as sterile when less than 20 progeny were present on a plate.

### smFISH

Single molecule RNA-FISH was performed as described in Lee C. S. et al. (2017), but germline dissections and all subsequent steps were performed on Poly-Lysine coated coverslips instead of test tubes to minimize loss of material. For dissection, worms were placed in a 15 μl drop of dissection buffer, while other steps were carried out in 30 μl of the corresponding buffer.

## Data Availability Statement

The data in this study have been deposited into the Gene Expression Omnibus (accession nos: GSM3684355, GSM3684356, GSM3684357, GSM3684358, GSM3684359, GSM3684360, GSM3684361, GSM3684362, GSM3684363, GSM3684364, GSM3684365, GSM3684366, and GSE128746).

## Author Contributions

VR, GY, SS, and FP: conceptualization. AR and GY: formal analysis. SS and FP: funding acquisition. VR, SG, and AK: investigations. VR, SG, GY, SS, and FP: methodology. VR, GY, and FP: writing – original draft preparation. SS, AK, and AR: editing. All authors contributed to the article and approved the submitted version.

## Conflict of Interest

The authors declare that the research was conducted in the absence of any commercial or financial relationships that could be construed as a potential conflict of interest.

## Footnotes

1 https://www.researchsquare.com/article/rs-17459/latest

2 www.r-project.org

3 http://pbil.univ-lyon1.fr/ADE-4/home.php?lang=eng

## Supplementary Material

FIGURE S1 Analysis of RNA-seq data. **(A)** Brood size of F2 to F7 single animals raised at 25°C. Each dot represents a single animal. A color code is used to show the F(n) animal from which the F(n + 1) animals were derived. **(B)** Normalized read counts in the RNA-seq experiments. **(C)** Two-dimensional density plot of 20,261 genes according to their maximal expression level (*X*-axis) and standard deviation (*Y*-axis) across all samples of the dataset. Each circle represents a gene. Gene density is represented as heat maps. The red vertical line delimits the set of 7,238 genes showing an expression value greater than 5 in at least one sample and analyzed by WCA.

FIGURE S2 Expression levels relative to P0 in F2, F4 fertile and F4 sterile for the top nine most downregulated contributors. The three independent lineages analyzed are represented by distinct line formats.

FIGURE S3 Expression levels relative to P0 in F2, F4 fertile and F4 sterile for the top nine most upregulated contributors. The three independent lineages analyzed are represented by distinct line formats.

FIGURE S4 Comparison of lists of contributor genes to list of genes misregulated in *set-2* mutant germlines at 20°C. ****p*-value < 0.001.

FIGURE S5 TGF-β components upregulation in *set-2* mutant germlines contributes to loss of identity. **(A)** Scheme of the TGF-β pathway present in *C. elegans*. Components upregulated during loss of germline identity are in red. **(B)** Expression relative to P0 of *daf-4*, *daf-14*, *daf-8*, *sma-4*, *daf-3*, *obr-3*, *kin-29, sma-9*, *daf-5*, and *sma-10* transcripts in F2, F4 fertile and F4 sterile. The three independent lineages analyzed are represented by distinctive line formats.

FIGURE S6 TGF-β components upregulation in *set-2* mutant germlines. **(A)** Detection by smFISH of *sma-3* transcript in P0 *set-2*/+ and F4 *set-2*/*set-2* germlines. Distal tip of the gonads are on the left. **(B)** Comparison of contributors with genes up- and downregulated in *sma-2* mutant animals. Statistical significance (hypergeometric test); **p* < 0.05, ***p* < 0.01, ****p* < 0.001. For downregulated contributors, *p* = 0.03 for under-enrichment in *sma-2* oocyte upregulated genes, *p* = 0.17 for over-enrichment in *sma-2* oocyte downregulated genes, *p* = 0.08 for under-enrichment in *sma-2* L4 upregulated genes, *p* = 0.001 for over-enrichment in *sma-2* L4 downregulated genes; for upregulated contributors: *p* = 5e-8 for over-enrichment in *sma-2* oocyte upregulated genes, *p* = 0.43 for under-enrichment in *sma-2* oocyte downregulated genes, *p* = 1.6e-32 for over-enrichment in *sma-2* L4 upregulated genes, *p* = 0.03 for under-enrichment in *sma-2* L4 downregulated genes. **(C)** Overlap between upregulated contributors and genes upregulated in *sma-2* oocytes or *sma-2* L4 stage animals. **(D)** Comparison of the list of 137 genes upregulated in *sma-2* oocytes or *sma-2* L4 stage animals and *set-2* mutant germlines with ubiquitous genes, soma specific genes (soma), germline enriched genes (germline), the wTF3.0 list of transcription factors (TF), CEBP-1 targets (CEBP-1), LIN-15B activated genes (LIN-15B), X-linked genes (X-linked), *mes-2*; *mes-4* upregulated genes (*mes* up) and GLP-1 activated genes (GLP-1). Soma specific genes (*p* = 2e-4) and LIN-15B activated genes (*p* = 5e-4) are significantly over-represented in this dataset. * *p*-value < 0.05, ** *p*-value < 0.01, *** *p*-value < 0.001.

TABLE S1 List of 7238 genes, downregulated and upregulated contributors with ubiquitous/germline/soma classification and comparison with genes deregulated following P-granule knock-down. Description of germline- specific downregulated contributors. Description of soma-specific upregulated contributors.

TABLE S2 Raw data of hypergeometric test performed on data presented on Figures 2, 3 and Supplementary Figures S4, S6.

TABLE S3 Comparison with transcription factors (wTF3.0), LIN-15B activated genes and CEBP-1 targets. Description of transcription factors found in upregulated and downregulated contributors.

TABLE S4 Comparison of genes misregulated in *mes-2*; *mes-4* mutants and genes activated by GLP-1/Notch and LIN-15B.

TABLE S5 RNAi targeting of positive contributors to the WCA PC1 in a *set-2*(*lf*) background (full list of tested genes).

TABLE S6 Comparison between downregulated contributors and TGF-β targets.
