# Supplementary figures and images for: Caenorhabditis elegans SET1/COMPASS Maintains Germline Identity by Preventing Transcriptional Deregulation Across Generations
Source: Front Cell Dev Biol. 2020 Sep 22;8:561791. doi: 10.3389/fcell.2020.561791 (PMC7536326; doi:10.3389/fcell.2020.561791)

A

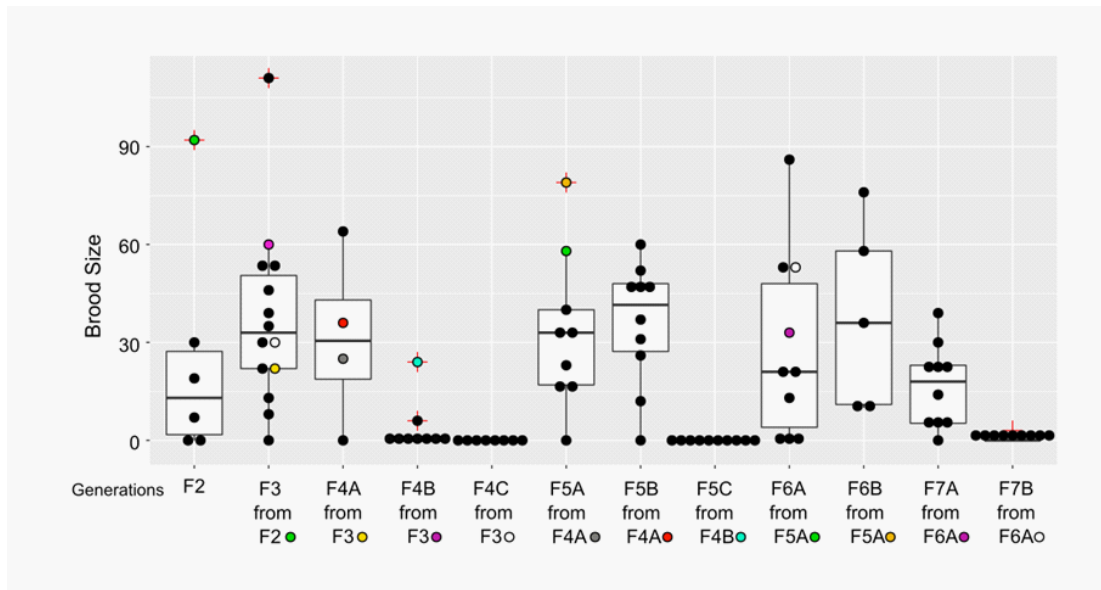

B

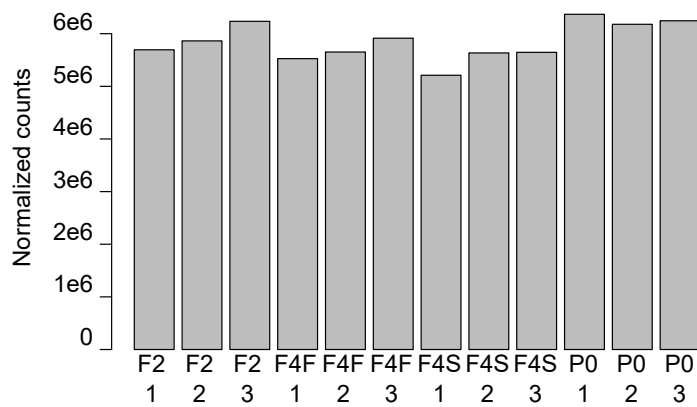

C

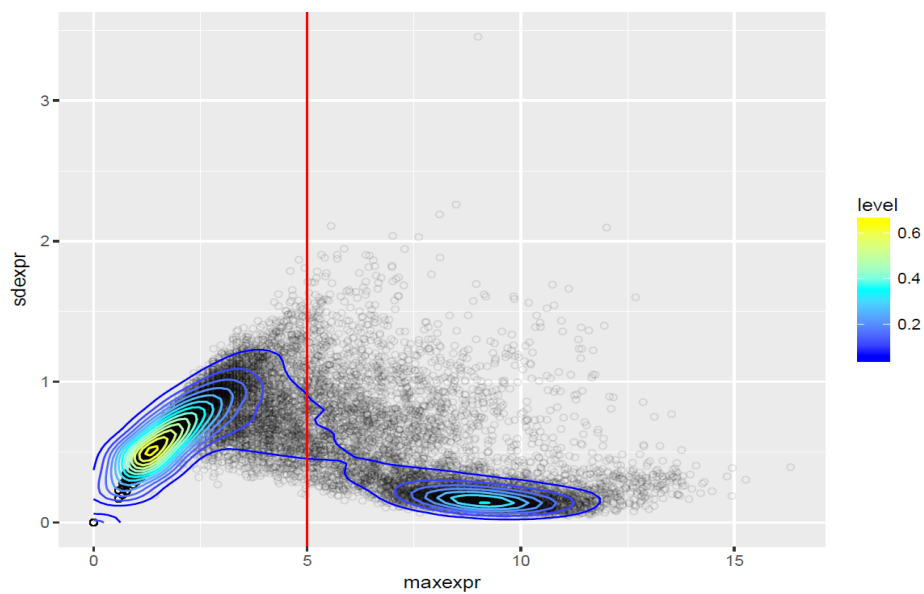

Supplement: FIGURE S1 — Analysis of RNA-seq data. (A) Brood size of F2 to F7 single animals raised at 25°C. Each dot represents a single animal. A color code is used to show the F(n) animal from which the F(n + 1) animals were derived. (B) Normalized read counts in the RNA-seq experiments. (C) Two-dimensional density plot of 20,261 genes according to their maximal expression level (X-axis) and standard deviation (Y-axis) across all samples of the dataset. Each circle represents a gene. Gene density is represented as heat maps. The red vertical line delimits the set of 7,238 genes showing an expression value greater than 5 in at least one sample and analyzed by WCA. [file Data_Sheet_1.PDF]

Figure S2

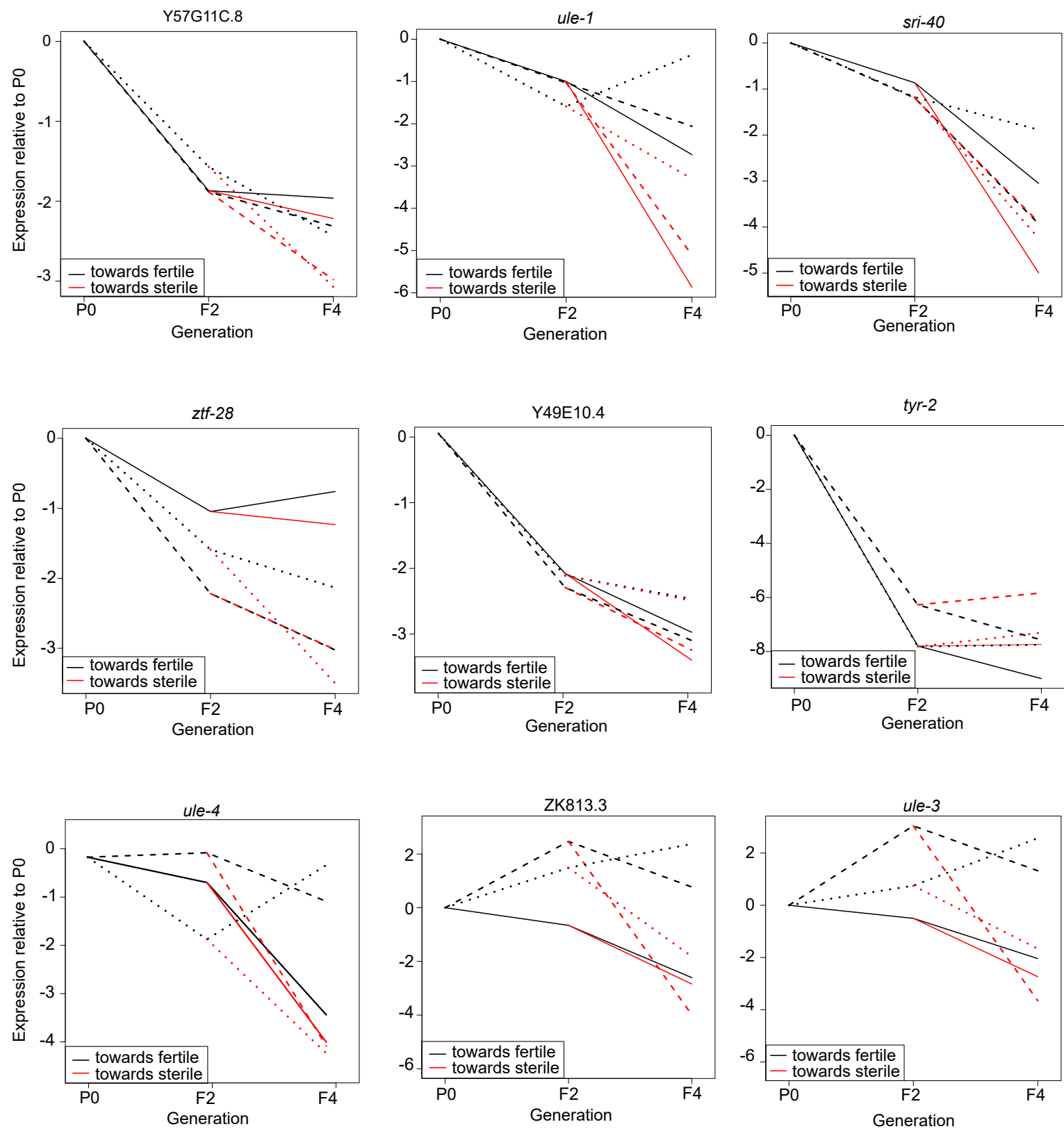

Supplement: FIGURE S2 — Expression levels relative to P0 in F2, F4 fertile and F4 sterile for the top nine most downregulated contributors. The three independent lineages analyzed are represented by distinct line formats. [file Data_Sheet_2.PDF]

Figure S3

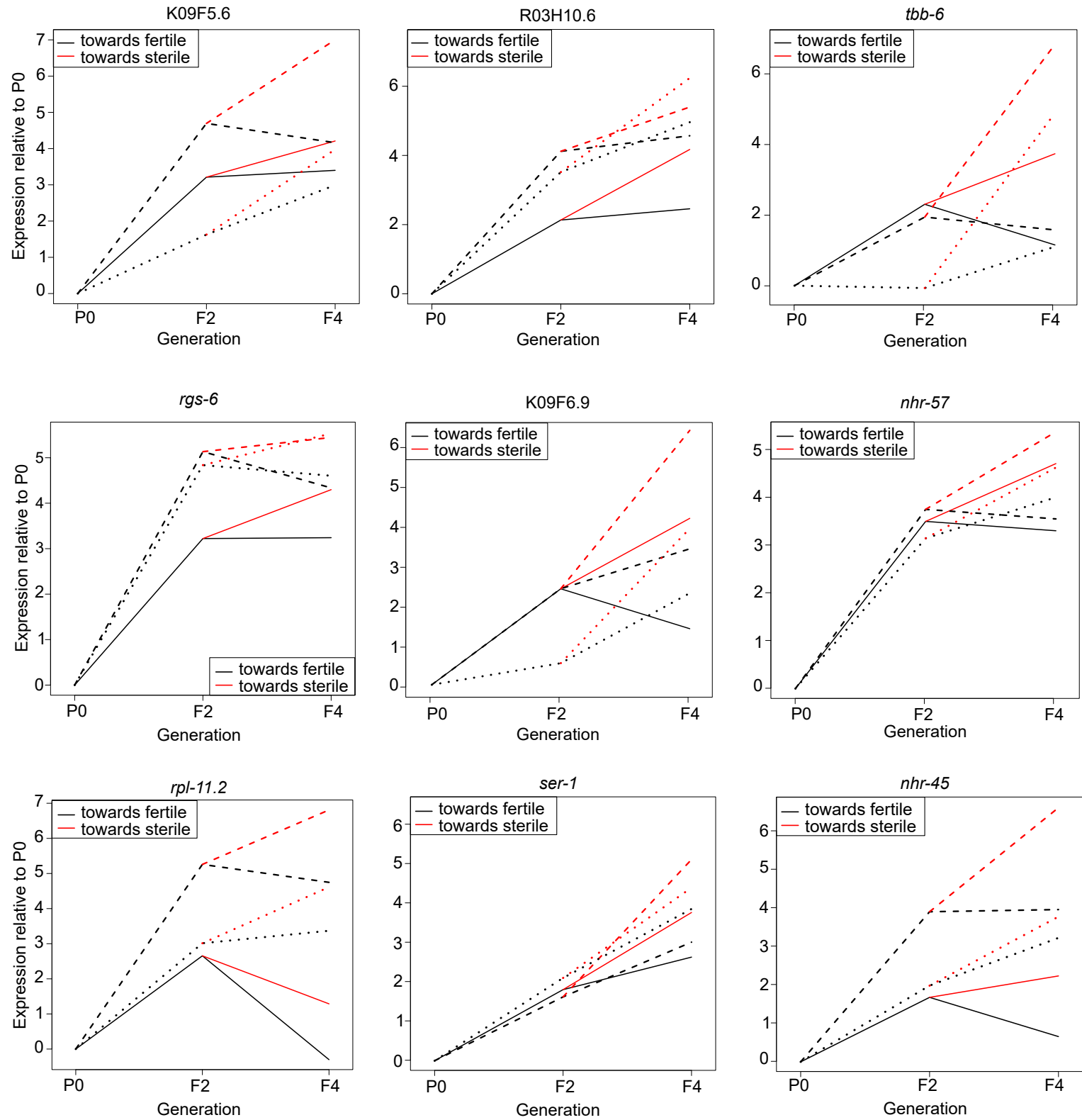

Supplement: FIGURE S3 — Expression levels relative to P0 in F2, F4 fertile and F4 sterile for the top nine most upregulated contributors. The three independent lineages analyzed are represented by distinct line formats. [file Data_Sheet_3.PDF]

Figure S4

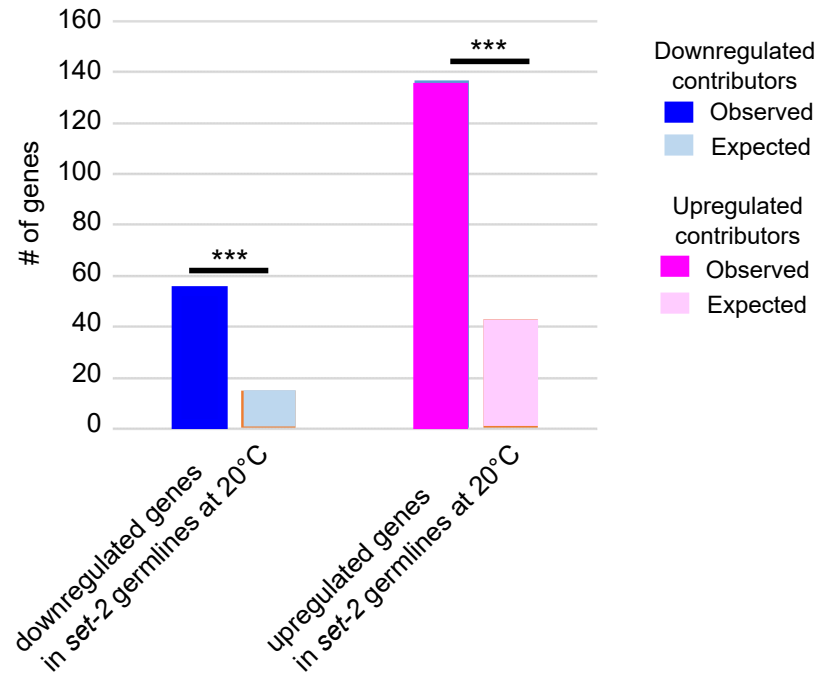

Supplement: FIGURE S4 — Comparison of lists of contributor genes to list of genes misregulated in set-2 mutant germlines at 20°C. ***p-value < 0.001. [file Data_Sheet_4.PDF]

Figure S5

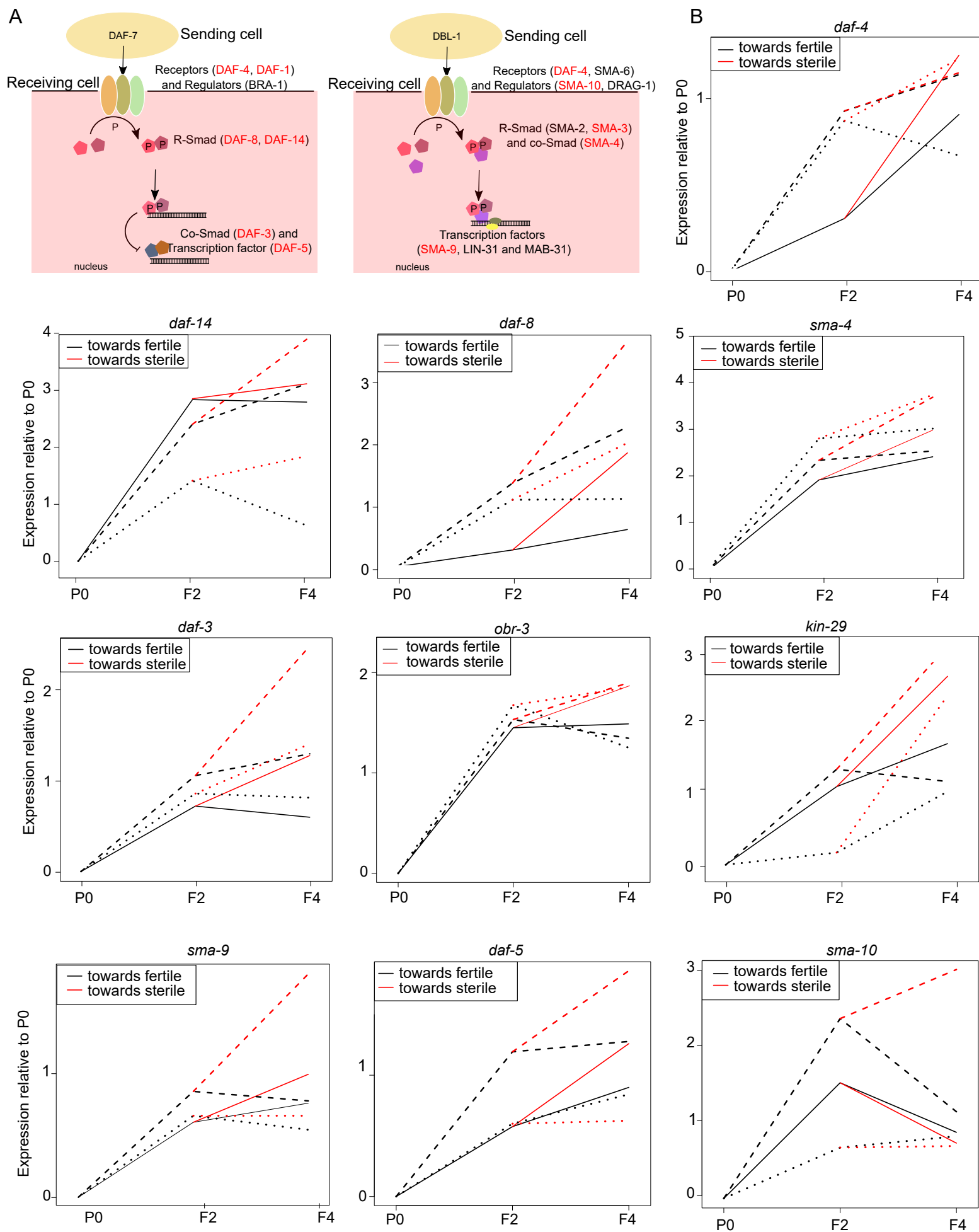

Supplement: FIGURE S5 — TGF-β components upregulation in set-2 mutant germlines contributes to loss of identity. (A) Scheme of the TGF-β pathway present in C. elegans. Components upregulated during loss of germline identity are in red. (B) Expression relative to P0 of daf-4, daf-14, daf-8, sma-4, daf-3, obr-3, kin-29, sma-9, daf-5, and sma-10 transcripts in F2, F4 fertile and F4 sterile. The three independent lineages analyzed are represented by distinctive line formats. [file Data_Sheet_5.PDF]

Figure S6

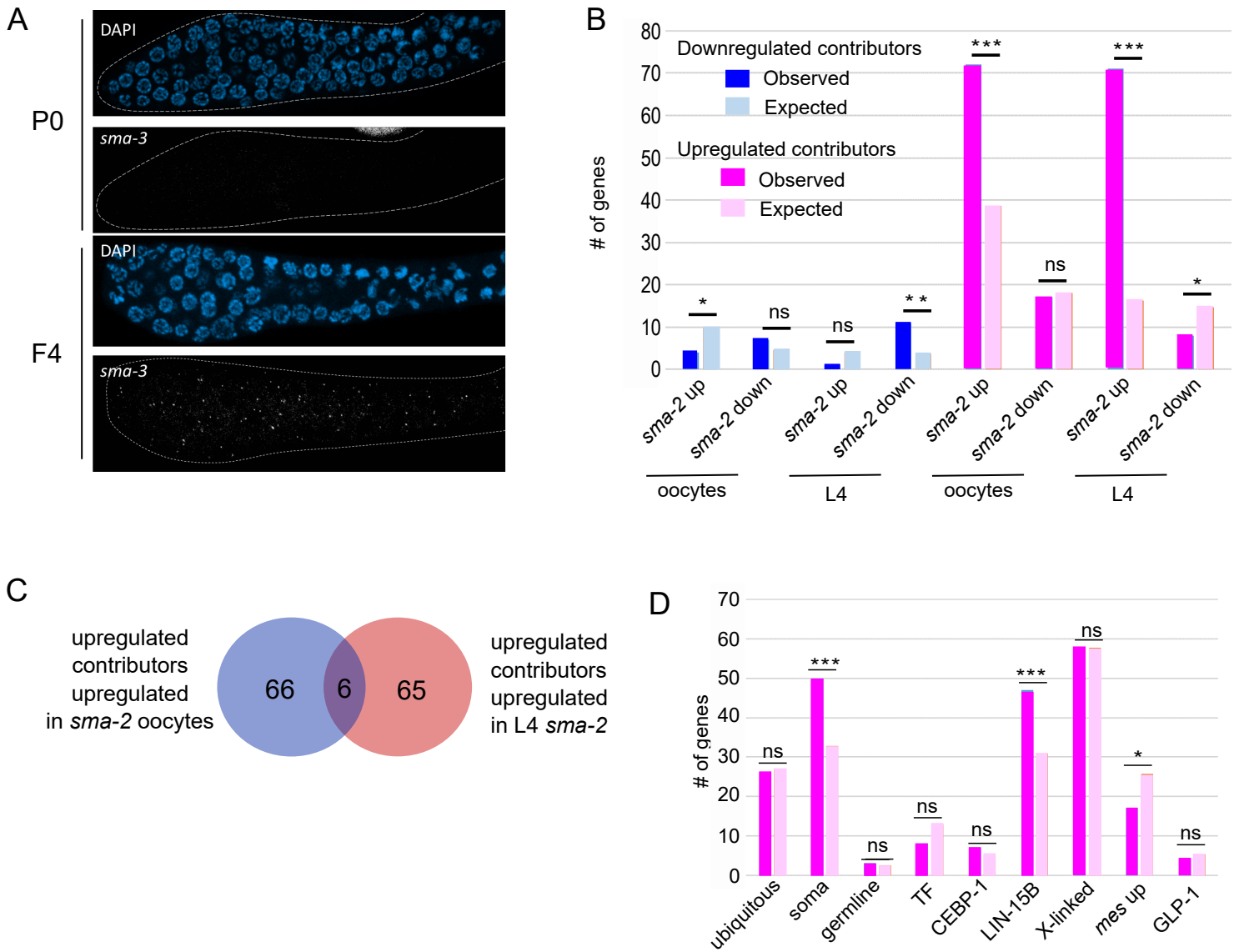

Supplement: FIGURE S6 — TGF-β components upregulation in set-2 mutant germlines. (A) Detection by smFISH of sma-3 transcript in P0 set-2/+ and F4 set-2/set-2 germlines. Distal tip of the gonads are on the left. (B) Comparison of contributors with genes up- and downregulated in sma-2 mutant animals. Statistical significance (hypergeometric test); *p < 0.05, **p < 0.01, ***p < 0.001. For downregulated contributors, p = 0.03 for under-enrichment in sma-2 oocyte upregulated genes, p = 0.17 for over-enrichment in sma-2 oocyte downregulated genes, p = 0.08 for under-enrichment in sma-2 L4 upregulated genes, p = 0.001 for over-enrichment in sma-2 L4 downregulated genes; for upregulated contributors: p = 5e-8 for over-enrichment in sma-2 oocyte upregulated genes, p = 0.43 for under-enrichment in sma-2 oocyte downregulated genes, p = 1.6e-32 for over-enrichment in sma-2 L4 upregulated genes, p = 0.03 for under-enrichment in sma-2 L4 downregulated genes. (C) Overlap between upregulated contributors and genes upregulated in sma-2 oocytes or sma-2 L4 stage animals. (D) Comparison of the list of 137 genes upregulated in sma-2 oocytes or sma-2 L4 stage animals and set-2 mutant germlines with ubiquitous genes, soma specific genes (soma), germline enriched genes (germline), the wTF3.0 list of transcription factors (TF), CEBP-1 targets (CEBP-1), LIN-15B activated genes (LIN-15B), X-linked genes (X-linked), mes-2; mes-4 upregulated genes (mes up) and GLP-1 activated genes (GLP-1). Soma specific genes (p = 2e-4) and LIN-15B activated genes (p = 5e-4) are significantly over-represented in this dataset. * p-value < 0.05, ** p-value < 0.01, *** p-value < 0.001. [file Data_Sheet_6.PDF]
