# Supplementary material for: Caenorhabditis elegans SET1/COMPASS Maintains Germline Identity by Preventing Transcriptional Deregulation Across Generations
Source: Front Cell Dev Biol. 2020 Sep 22;8:561791. doi: 10.3389/fcell.2020.561791 (PMC7536326; doi:10.3389/fcell.2020.561791)
Supplement: TABLE S5 — RNAi targeting of positive contributors to the WCA PC1 in a set-2(lf) background (full list of tested genes). [file Table_5.DOCX]

|  | Functional class | chr | Generations | | |
| --- | --- | --- | --- | --- | --- |
|  |  |  | F4 | F8 | F12 |
| A | Transcription |  |  |  |  |
|  | *cebp-1* | X | + | + | + |
|  | *daf-19* | II | - | + | + |
|  | *attf-5* | X | - | + | + |
|  | *somi-1* | V | - | + | +/- |
|  | *flh-2* | III | - | - | - |
|  | *gfi-3* | X | - | - | - |
|  | *nhr-48* | X | - | - | - |
|  |  |  |  |  |  |
|  | Chromatin |  |  |  |  |
|  | *jmjd-3.1* | X | - | - | - |
|  | *utx-1* | X | - | - | - |
|  |  |  |  |  |  |
|  | Phospho. |  |  |  |  |
|  | *pek-1* | X | - | + | + |
|  | *kgb-1* | IV | + | - | - |
|  |  |  |  |  |  |
|  | Others |  |  |  |  |
|  | *avr-14* | I | + | + | + |
|  | *crb-1* | X | + | + | + |
|  | *aldo-1* | III | - | + | + |
|  | *fhod-1* | I | - | - | + |
|  | *puf-9* | X | - | + | - |
|  | *miz-1* | IV | - | - | - |
|  | *ncam-1* | X | - | - | - |
| B | TGF beta |  |  |  |  |
|  | *daf-5* | II | + | + | + |
|  | *kin-29* | X | + | + | + |
|  | *obr-3* | X | + | +/- | + |
|  | *sma-3* | III | - | + | + |
|  | *sma-9* | X | - | + | + |
|  | *sma-10* | IV | - | + | + |

Table S5: RNAi screening for delay of the onset of sterility in a *set-2*(*lf*) background. (A) Assay for upregulated contributors including transcription and chromatin factors, phosphatases and others. RNAi knock-down of *his-8*, *ceh-43*, *hbl-1*, *lin-59*, *nhr-57*, *sop-2*, *sor-1,* *ckk-1,* *eef-1A.2*, *glp-1,* and *rpl-11.2* resulted in early sterility that prevented further investigation of their role (data not shown). (B) Assay for components of the TGF-β signaling pathway. Number of progeny was estimated in F4, F8 and F12 generations. ‘-‘ indicates that number of progeny is equivalent to the one observed with the L4440 control food. ‘+’ indicates that number of progeny is greater to what is observed with the L4440 control food.
